# The function and regulatory mechanism of RNA-binding proteins in breast cancer and their future clinical treatment prospects

**DOI:** 10.3389/fonc.2022.929037

**Published:** 2022-08-16

**Authors:** Xingjia Lu, Jian Zhong, Linlin Liu, Wenzhu Zhang, Shengdi Zhao, Liang Chen, Yuxian Wei, Hong Zhang, Jingxuan Wu, Wenlin Chen, Fei Ge

**Affiliations:** ^1^ Department of Breast Surgery, First Affiliated Hospital of Kunming Medical University, Kunming, China; ^2^ Kunming Medical University, No. 1 School of Clinical Medicine, Kunming, China; ^3^ Department of Reproductive Medicine, Affiliated Jinling Hospital, Nanjing Medical University, Nanjing, China; ^4^ Department of Gynecology, Women’s Hospital of Nanjing Medical University, Nanjing, China; ^5^ School of Forensic Medicine, Kunming Medical University, Kunming, China; ^6^ Department of Endocrine Breast Surgery, First Affiliated Hospital of Chongqing Medical University, Chongqing, China; ^7^ Third Department of Breast Surgery, The Third Affiliated Hospital of Kunming Medical University, Kunming, China

**Keywords:** breast cancer, RNA-binding protein, HuR, LIN28, Sam68, CPEB4

## Abstract

Breast cancer is the most common female malignancy, but the mechanisms regulating gene expression leading to its development are complex. In recent years, as epigenetic research has intensified, RNA-binding proteins (RBPs) have been identified as a class of posttranscriptional regulators that can participate in regulating gene expression through the regulation of RNA stabilization and degradation, intracellular localization, alternative splicing and alternative polyadenylation, and translational control. RBPs play an important role in the development of normal mammary glands and breast cancer. Functional inactivation or abnormal expression of RBPs may be closely associated with breast cancer development. In this review, we focus on the function and regulatory mechanisms of RBPs in breast cancer, as well as the advantages and challenges of RBPs as potential diagnostic and therapeutic targets in breast cancer, and discuss the potential of RBPs in clinical treatment.

## Introduction

Breast cancer is a highly prevalent malignancy worldwide and is the most common cause of cancer death in women in particular (1).In recent years, the incidence of breast cancer has increased at a rate of 0.5% per year. The reason for this increase is the continued decline in fertility and weight gain, so the global incidence of female breast cancer is predicted to be as high as 3.2 million cases per year by the year 2050 (2, 3). In terms of historical classification, to a large extent, breast carcinogenesis is based on the oncogenic activity of estrogen receptor α (ERα) as well as other hormone receptors, progesterone receptor (PR) and human epidermal growth factor receptor 2(HER2/ERBB2). Based on the expression of these proteins, breast cancers are classically classified into five subtypes: luminal A (ER+, PR+, HER2-), luminal B (ER+, PR-, HER2+), HER2-positive (ER-, PR-, HER2+), basal-like and triple-negative breast cancers (ER-, PR- and HER2-), while the last two subtypes are similar but distinct from invasive breast cancer (4).Currently, the treatment strategies for breast cancer are determined mainly based on tumor size, morphology, metastasis and expression of ER, PR, Ki67 and HER2, including surgery, radiotherapy, endocrine therapy and chemotherapy, which have led to a great delay in tumor progression and further improvement in patient survival (5).However, these therapeutic strategies have not been clinically effective, so there is an urgent need to explore additional molecular regulatory mechanisms of breast cancer to develop new diagnostic and therapeutic targets.

**Figure 1:**
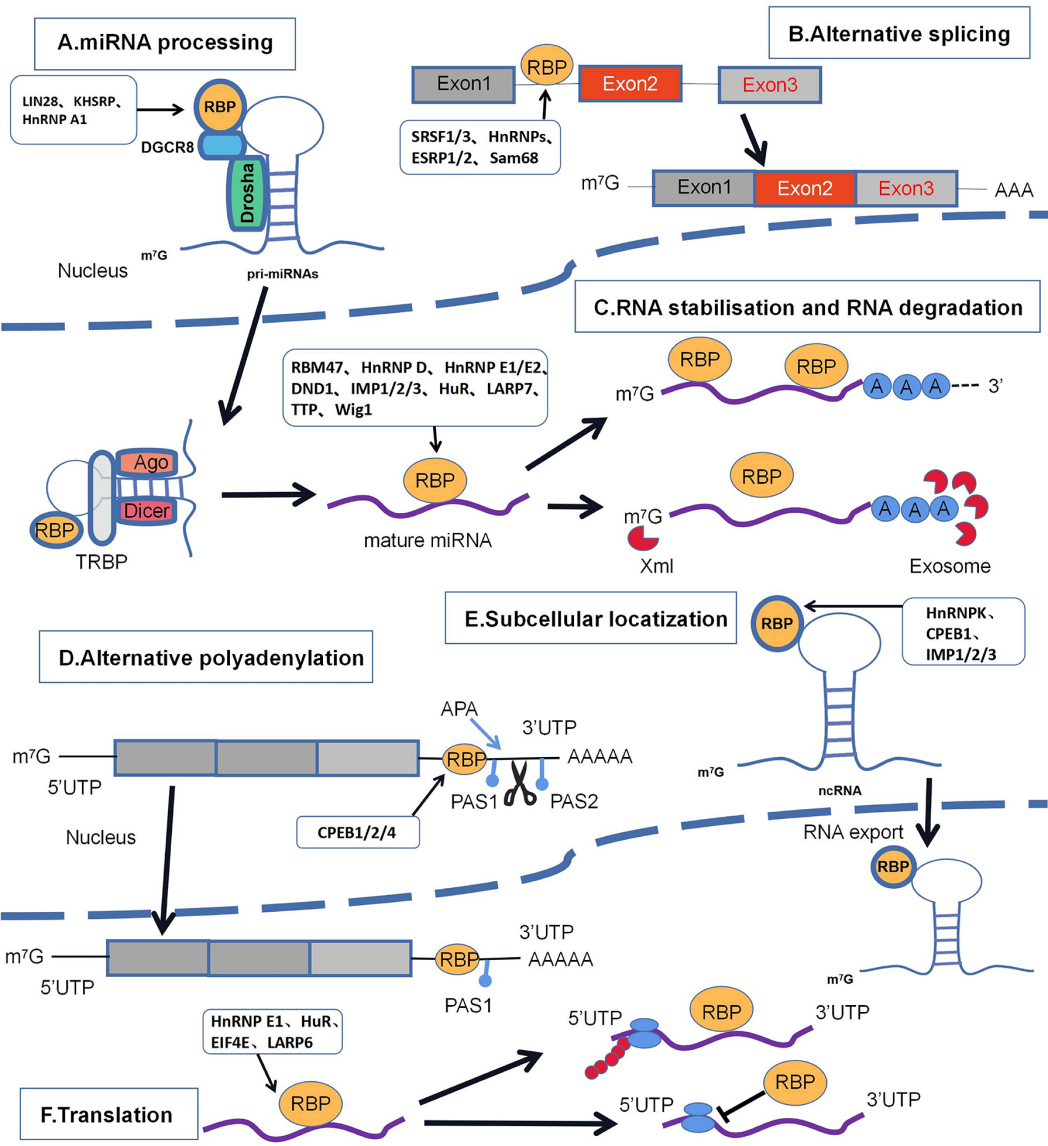
Major regulatory mechanisms of RBPs in breast cancer. including **(A)** miRNA processing; **(B)** selective splicing; **(C)** RNA stabilization and RNA degradation; **(D)** selective polyadenylation; **(E)** subcellular localization; **(F)** translation. The schematic diagram lists the RBPs involved in the regulatory mechanisms of breast cancer that appear in the article.

RNA-binding proteins (RBPs) bind to various types of RNAs through RNA-binding domains (RBDs), resulting in stable secondary and tertiary structures of RNA. The K-homology structural domain (KH), RNA recognition motif (RRM), zinc finger structural domain (ZNF), PUM structural domain (PUM), and double-stranded RNA binding structural domain (DSRBD) are the classical RBDs (6, 7). Specifically, RBPs can recognize and interact with RNA recognition motifs (RRMs) and/or binding motifs of RNA structures to form ribonucleoprotein (RNP) complexes to regulate RNAs through, for example, microRNA (MiRNA) processing, RNA stability, alternative premRNA splicing, mRNA decay, translocation, posttranslational nucleotide modifications, and RNA localization (**Figure 1**) (8, 9).Therefore, RBPs play a key role in the regulation of gene expression at the posttranscriptional level. Dysregulated gene expression of some RBPs may lead to the development of various diseases, including cancer (10).With the in-depth study of gene regulation in breast cancer, it has been found that some RBPs in breast cancer are functionally inactivated or have altered expression. Therefore, it is urgent to explore the function and mechanism of RBPs in breast cancer development.

In this review, we will discuss the function of RBPs in breast cancer cells and their regulatory mechanisms, as well as their potential targets for diagnosis and treatment, providing new therapeutic strategies for the future.

## Mechanism of RBPs in breast cancer

In recent years, the specific expression and function of RBPs in breast cancer can be revealed using advanced bioinformatics tools, such as analysis of RNA-seq data based on The Cancer Genome Atlas (TCGA) data. The results of GO and KEGG analysis showed that these RBPs up- or down-regulated in breast cancer are mainly involved in RNA processing, splicing, localization and RNA silencing, and transcriptional regulation.In addition, there are RBPs associated with estrogen response, inflammatory mediators and translational regulation, which in turn are involved in the process of breast cancer development, invasion and metastasis. A recent study showed that 90 RBPs were upregulated and 115 RBPs were downregulated in breast cancer (11). Herein, we review the main regulatory mechanisms of RBPs in and how their dysregulation leads to the development of breast cancer (**Table 1**).

**Table 1:** Roles of RNA binding proteins (RBPs) in breast cancer.

RBP	Expression	Mechanisms	Targets	Traits	References
LIN28A/B	Upregulated	miRNA processing	let-7	Proliferation, invasion, metastasis, angiogenesis	(12–18)
KHSRP	Upregulated or downregulated	miRNA processing	miR-192-5p, let-7	EMT, invasion, metastasis	(19, 20)
HnRNP1	Upregulated	miRNA processing, Alternative splicing	miR-18a, let-7a, RON, caspase-2	Proliferation, EMT	(21–25)
HnRNPD(AUF1)	Upregulated	mRNA stability	c-Yes, Cyclin D1, MMP9, Myc	Proliferation, Senescence	(26, 27)
HnRNPE1/2 (PCBP1/2)	Upregulated or downregulated	mRNA stability, translation	p27, UFD1, NT5E, ILEI	Senescence, EMT, invasion, metastasis	(28–33)
HnRNP M	Upregulated	Alternative splicing	CD44	EMT, invasion, metastasis	(25, 34)
HnRNP I(PTB)	Upregulated	Alternative splicing	FGFR-1, USP5, PKM, Cyclin D3	Proliferation	(21, 35)
HnRNP H1	Upregulated or downregulated	Alternative splicing	MADD30, Bcl-xs △16HER2	Proliferation	(36)
HnRNP K	Upregulated	Subcellular localization	c-myc, lncRNA MLXIPL	Metastasis, proliferation	(37, 38)
SRSF1(SF2/ASF)	Upregulated	Alternative splicing	BIM, BIN1	Senescence, EMT, invasion, metastasis, proliferation, angiogenesis	(25, 39)
SRSF3 (SRp20)	Upregulated	Alternative splicing	FoxM1, GR	Proliferation, apoptosis, EMT, metastasis	(40, 41)
SRP 1/2	Upregulated	Alternative splicing	Rac1, CD44, E-cadherin	EMT, invasion, metastasis	(42, 43)
Sam68	Upregulated	Alternative splicing	CD44v5, Cyclin D1, Bcl-xs	Proliferation, EMT, invasion, metastasis	(44–46)
RBM47	Downregulated	mRNA stability	Dkk1	Metastasis	(47)
DND1	Downregulated	mRNA stability	BIM	Apoptosis	(48)
IGF2BP1 (IMP1/ZBP1)	Upregulated	mRNA stability, Subcellular localization	β-catenin E-cadherin lncRNA UCA1	Proliferation, EMT, invasion, metastasis	(49–52)
IGFBP2(IMP2)	Upregulated	mRNA stability	E-cadherin, PR miR-200a	EMT, invasion, metastasis	(53)
IGF2BP3(IMP3)	Upregulated	mRNA stability	PR, miR-200a	Proliferation, EMT, invasion, metastasis	(53)
HuR	Upregulated or downregulated	mRNA stability, translation	p21, CDK1, CDK7, VEGF-A, MMP9, ER, IL-8, calmodulin HOX-A5, CD9, FOXO1, erbB2, CXCR-4, SiRT1, SOCS3, HIF-1-α, Wnt5a, TP63, BRCA1, IGF1R, miR-125b	Proliferation, apoptosis, angiogenesis,senescence, invasion, metastasis	(54–56)
LARP6	Upregulated	Translation	MMP-9, VEGF	Angiogenesis, EMT, Proliferation, invasion	(57, 58)
LARP7	Downregulated	mRNA stability	FOXC2,Slug,Twist1, ZEB2, 7SK snRNP	EMT, invasion, metastasis	(59, 60)
TTP	Upregulated or downregulated	mRNA stability	Cyclin B1,Cyclin D1, Bcl-2, VEGF	Angiogenesis, metastasis senescence, Proliferation	(61, 62)
Wig1 (ZMAT3)	Downregulated	mRNA stability	p53	Senescence	(63–65)
CPEB1	Upregulated	Alternative Polyadenylation, Subcellular localization	MMP9 ZO-1	Proliferation, invasion, angiogenesis, metastasis, EMT,	(66)
EIF4E	Upregulated	Translation	c-Myc, Cyclin D1	Apoptosis, angiogenesis, EMT, invasion, metastasis	(67–70)

### Dysregulation of miRNA processing of RBPs may contribute to breast cancer development

RBPs are key regulators that control the different stages of miRNA biogenesis and maturation, as well as their localization, degradation and activity, and they promote or inhibit miRNA processing mainly through their action on canonical proteins (such as DROSHA and Disher). In recent years, studies have shown that RBPs play an important role in miRNA processing and function; therefore, dysfunction and altered expression levels of RBPs are associated with miRNA processing disorders leading to the dysregulation of target mRNAs, which contribute to tumorigenesis and development of breast cancer (21, 71).

LIN28 (LIN28a and LIN28b) is known to be one of the RBPs with two RNA binding motifs: the cold shock structural domain(CSD) and the Cys-Cys-His-Cys(CCHC) zinc finger structural domain (12). These structural domains of LIN28 are required for direct interaction with the terminal loop (TL) of pre-let-7, thereby inhibiting the biogenesis of let-7 miRNAs (13). It has been reported that LIN28 is mainly localized in the cytoplasm, and LIN28 specifically binds to the terminal loop region of pre-let-7 miRNA, which isolates pri-miRNAs in the cytoplasm and acts as a distraction from the nuclear microprocessor complex, ultimately inhibiting miRNA processing (14). The family of let-7 microRNAs (miRNAs) is a key inhibitory target of LIN28 and exerts potent tumor suppression through posttranscriptional inhibition of multiple oncogenic messenger RNAs (mRNAs) (15). Research has shown that the most fundamental feature of LIN28 in breast cancer cells is its ability to promote and maintain slow proliferation. For example, LIN28 achieves direct or indirect regulation of let-7 by repressing let-7 to enable it to function as an oncogene, including the dysregulation of several genes that are components of the MYC, HMGA2, and PI3K-mTOR pathways (16). The reduction of let 7 mediated by LIN28 downregulates let-7 target genes, leading to abnormalities in the LIN28/let-7 pathway and contributing to tumor proliferation, invasion, metastasis, inflammation, and angiogenesis (17, 18).

KH-type splicing regulatory protein (KHSRP) is a single-stranded multifunctional RNA-binding protein that is involved in posttranscriptional aspects of RNA metabolism and plays an important role in the development of breast cancer (72, 73). KSRP, a component of the DROSHA and DICER complex, is able to regulate the biogenesis of a portion of miRNAs and is also a key regulator involved in miRNA precursor processing due to the high affinity of KSRP for the terminal loop (TL) of target miRNA precursors and promotes the maturation of miRNAs (19). KHSRP is a key factor in maintaining the epithelial phenotype, which facilitates mRNA decline and miRNA maturation. For example, KHSRP in NMuMg cells (a mouse immortalized mammary epithelial cell line) promotes maturation of precursor miR-192-5p, which upregulates EMT factor expression. In contrast, the expression of anti-miR-192-5p in NMuMg cells upregulates the expression of Zeb1, ZEB2, Snai1, Iglon5, and Mmp9 but does not affect the mRNA levels of FSTL1, even leading to the downregulation of the expression of EMT factors, such as Fn1, Col6a2, and Col12a1 (20).

Some specific RNA-binding proteins (RBPs) have emerged as important posttranscriptional regulators of miRNA processing, such as our discovery of heteronuclear ribonucleoprotein A1 (HnRNP A1), a cofactor containing two RRM structural domains that can make specific contacts through the terminal loop of RNA. Subsequently, processing of miRNA precursors can begin, such as the regulation of miRNA-18a (pri-mir-18a) processing, which mainly binds specifically to two UAG motifs of pri-miR-18a (one in the TL and one in the proximal stem region), forms a 1:1 complex with this miRNA, and relaxes the pri-miRNA stem, thus improving the cleavage efficiency of DROSHA (21, 22). When miR-18a expression is reduced, SREBP1 overexpression occurs, E-cadherin is suppressed, Snail/HDAC1/2 complex formation occurs, and EMT is ultimately induced in breast cancer cells (23). HnRNP A1 can also act as a negative regulator of let-7a processing, competing with the activator protein KHSRP for the pri-let-7a terminal loop, leading to a block in the interaction of KHSRP and thus increasing let-7a biogenesis, so HnRNP A1 and KHSRP have an antagonistic role in the posttranscriptional regulation of let-7 precursor processing (22, 24).

### RBPs, as splicing factors, regulate alternative splicing to influence the related process of breast cancer

Alternative splicing is one of the most prevalent functions of RBPs in gene regulation. RNA splicing is a form of RNA processing in which newly generated precursor messenger RNA (premRNA) transcripts are converted into mature messenger RNA (mRNA) (74). Specifically, alternative splicing is the process of rearranging exon, partial exon, and/or partial intron combinations into mature RNAs by selecting different combinations of premRNAs from different regions to form different mature mRNAs, thus achieving genetic diversity (25, 75, 76). The splicing process is a sequential phosphodiester transfer reaction catalyzed by a large ribonucleoprotein complex composed of the small nuclear ribonucleoproteins (snRNPs) U1, U2, U4, U5 and U6 and splicing factors (which are RNA-binding proteins targeting specific RNA sequences or motifs) (25). Studies have shown that splicing factors play a dual role in activating or inhibiting splicing events, and once these RBPs bind to pre-RNAs, they can either facilitate or block the interaction between spliceosomes and premRNAs (25). Therefore, abnormalities in alternative splicing may systematically affect all cancer-related processes, such as epithelial-to-mesenchymal transition (EMT) (77).

Serine/arginine-rich splicing factors (SRS) belong to a family of serine-rich proteins, typically consisting of 12 members (SRSF1-12), that play a key role in controlling alternative splicing in cancer, for which aberrant expression of SRS, for example, leads to aberrant RNA splicing and ultimately affects tumor cell proliferation, migration and apoptosis (78). One study found that the SR proteins SRSF1, SRSF2, SRSF3, SRSF5 and SRSF6 are overexpressed in breast cancer (25). Overexpression of SRSF1 inhibits apoptosis and promotes the transformation of mammary epithelial cells by inducing alternative splicing of the antiapoptotic splice isoforms BIM and BIN1 and the expression of splice variants lacking the BH3 structural domain (39). SRSF3 is the smallest SR protein involved in the alternative splicing of FoxM1, producing FoxM1a, b and c1a isoforms (40). During alternative splicing, SRSR3 recognizes the CUC(U/G)UCY splice enhancer sequence, a process promoted by the N6-methyladenosine (m6A) reader protein YTH structural domain containing 1 (YTHDC1), which in turn prevents binding of SRSF10 mRNA and ultimately promotes exon inclusion of the target mRNA (79). It has been shown that SRSF3-induced expression promotes the splicing of glucocorticoid receptor (GR) to GRα, which upregulates activated C-kinase receptor 1 (RACK1) and leads to a significant increase in MDA-MB-231 cell migration. In contrast, silencing RACK1 or SRSF3 prevents this increase (41).

The splicing factor heterogeneous ribonucleoproteins (HnRNPs) are a family of RNA-binding proteins (RBPs) containing at least 20 members with a common structural domain that positively or negatively control splicing by binding to different regions of premRNA (80). In addition, SR proteins typically compete with splicing factors (HnRNPs) to block entry of spliceosome elements by binding to exon or intron splice silencing factors (ESSS or ISSS) and result in inhibition of splice site selection. SR proteins that act as antagonists of HnRNPs in a concentration-dependent manner can prevent exon skipping (81). The HnRNP family members HnRNPA1, HnRNPA2, HnRNPI, HnRNPM and HnRNPK have been reported to be highly expressed in breast cancer (25). In particular, HnRNPA1 not only reduces the formation of the EMT-driven isoform ΔRON by producing a tumorigenic splice variant of RON but also acts as an oncoprotein that promotes the inclusion of exon 9 of the tumor suppressor caspase-2, resulting in the production of the truncated antiapoptotic isoform caspase-2S (25). Binding to the GC-rich structural domain of CD44, HnRNPM promotes the skipping of exon 8, which ultimately promotes breast cancer metastasis by enhancing TGFβ signaling and thus activating the switch of alternative splicing that occurs during epithelial-mesenchymal transition (EMT) (25, 34). Known as polypyrimidine domain binding protein (PTB), HnRNPI functions as a splicing repressor, regulating cancer-associated alternative splicing events by interacting with pyrimidine-rich sequences, such as exon skipping or inclusion when PTB is knocked down (35).

Two splicing factors, HnRNP H1 and SRSF3, involved in the regulation of splicing in highly spliced regions were found to be present in HER2-overexpressing breast cancers by RNA interference experiments. However, the role of HnRNP H1 in cancer development is still complicated by its ability to upregulate anti-apoptotic heterodimers (MADD30) and pro-apoptotic spliceosomes (Bcl-xS), such as the increase in the oncogene Δ16HER2 variant observed following knockdown of HnRNP H1, suggesting that deletion of this splicing factor may lead to a more oncogenic phenotype (36).

ESRP1 and ESRP2 belong to the RNA-binding protein RBM family, also known as RBM35A and RBM35B, respectively, and are epithelial-specific splicing regulators that control the splicing process of epithelial-to-mesenchymal transition (EMT) in cancer. It has been found that knockdown of ESRP1 increases the expression of Rac1b isoforms by allowing alternative splicing of Rac1 mRNA to include variant exon 3b, while in ESRP1 knockdown cells, Rac1b regulates actin dynamics, increases cell motility and induces the formation of long filamentous pseudopods (42). It was shown that ESRP1 promotes lung cancer metastasis by regulating CD44 splicing in ER-negative 4T1 mouse mammary tumor cells. In addition, overexpression of ESRP1 and ESRP2 in basal-like breast cancer cells resulted in upregulation of E-cadherin expression, while in an ER-negative breast cancer model (MDA-MB-231 cells), low ESRP1 expression was associated with the development of EMT. In contrast, ESRP1 drove invasiveness in ER+ breast cancers independent of EMT, and thus, high ESRP1 expression but not ESRP2 was significantly associated with reduced overall survival in breast cancer patients as well as with poor prognosis in ER+ breast cancers, suggesting that the malignant phenotype of human breast cancer is associated with ESRP1 overexpression (42, 43).

Sam68 (68 kDa SRC-associated substrate during mitosis), which belongs to the STAR (signal transducer and RNA activator) RNA-binding protein family, is the first BRK phosphorylated substrate identified *in vivo* and promotes cell growth mainly by regulating alternative mRNA splicing. Sam68 regulates CD44v5, cyclinD1 and Bcl-xs mRNA splicing (44, 45). In living cells, Sam68, when phosphorylated by Src-like kinase, alters the splicing of Bcl-x and leads to the ratio change of the two splice variants it encodes, pro-apoptotic Bcl-x(S) and anti-apoptotic Bcl-x(L), which facilitates the accumulation of Bcl-x(L) and thus keeps cancer cells from undergoing apoptosis (82). Sam68 is significantly overexpressed in breast cancer cells and tissues and is associated with shorter survival rates; conversely, downregulation of endogenous Sam68 expression leads to suppression of proliferation and tumorigenicity of breast cancer cells (46).

### RBPs maintain RNA stability by binding to the mRNA 3’UTR and thus affect breast cancer

One of the determinants of RNA stability is the 5’7-methylguanine nucleoside cap, which is bound together by cotranscription factors to prevent mRNA decline and facilitate translation initiation. Conversely, the well-known regulatory pathway of mRNA is the 3’ end of polyadenosine. After transcription, a group of terminal nucleotidyl transferases (Tents) called poly(A) polymerases (PAPs) add untemplated adenosine residues to the 3’ end of the transcript to stabilize the mRNA by interacting with poly(A)-binding proteins (PAMPs) (83, 84).

RNA-binding motifs (RBMs) are novel RBPs with one or more RNA recognition motif (RRM) structural domains, of which RBM47 has three RRM structural domains that can play an important role as tumor suppressors in posttranscriptional regulation, mainly by inhibiting EMT and Wnt/β-catenin signaling (85). Low RBM47 expression is significantly associated with a poor prognosis in two subtypes of claudin-low breast cancer and basal breast cancer. In addition, RBM47 binds mainly to the intron and 3’UTR of the target mRNA, with the strongest binding occurring in the 3’UTR (47). RBM47 increases the stability of Dkk1 mRNA in breast cancer cells through direct binding to the noncoding region at the 3’ end of Dkk1 mRNA. Dkk1 is a secreted protein that suppresses tumor metastasis and is also an inhibitor of Wnt signaling, which has been shown to promote breast cancer progression. RBM47 can increase Dkk1 secretion, which in turn inhibits Wnt signaling, thereby reducing the tumorigenic fitness of metastatic breast cancer cells (47). As a result, RBM47 inhibits the progression and metastasis of breast cancer.

Heterogeneous ribonucleoprotein D (HnRNP D), also known as Au-rich element RNA binding protein 1 (AUF1), is localized to the 3’ untranslated region (3’UTR) of many unstable mRNAs and consists of four different protein isoforms: p40^AUF1^ and p37^AUF1^ are commonly found in the cytoplasm and nucleus, whereas the P45^AUF1^ and p42^AUF1^ isoforms are predominantly found in the nucleus (26). These isoforms have a high affinity for unstable sequences of mRNA and AU-rich (AREs) sequences located in the 3’UTR of mRNAs, and therefore, HnRNP D promotes mRNA decline through ARE-mediated decline (AMD) (26, 80). c-Yes is a member of the c-Src family of tyrosine kinases. In MDA-MB-231 human breast cancer cells, downregulation of c-Yes expression levels leads to overexpression of the small molecule heat shock protein 27 (Hsp27), immediately followed by increased invasive ability *in vitro* and metastatic behavior *in vivo (* 27). The expression regulation of c-Yes may be mediated by regulatory sequences in the 3’UTR because the c-Yes 3’-UTR can interact with AUF1 and HuR, which may accelerate mRNA degradation, ultimately leading to the downregulation of c-Yes (27).

The HnRNPs E1 and E2, also commonly referred to as poly(C)-binding proteins PCBP1 and PCBP2, are composed of HnRNP K/J and HnRNP K homology structural domain (KH) alpha-complex proteins (CP1-4 or PCBP1-4α) (28). PCBP1 stabilizes p27 mRNA mainly by binding to the p27 3’UTR through its Kh1 structural domain, which enhances its translation, promotes p27 protein expression, induces cell cycle arrest, inhibits cell proliferation, and ultimately suppresses tumorigenesis *in vitro* and *in vivo*. Conversely, knockdown of PCBP1 in turn accelerates p27 mRNA degradation, causes low p27 (cell cycle inhibitor) protein levels and leads to the development of breast cancer. It has been reported that PCBP1 expression is downregulated in breast cancer (29). In addition, both UFD1 and NT5E knockdown inhibit cell proliferation, colon formation, migration and invasion in breast cancer. Overexpression of PCBP2 promotes the proliferation and metastasis of breast cancer cells by maintaining the mRNA stability of UFD1 and NT5E. PCBP2 binds to the 3’UTR of UFD1 and NT5E to upregulate the expression of these two downstream genes, which ultimately promotes the development of breast cancer (30).

The RNA binding protein DND1 is an evolutionarily conserved RBP that maintains the stability of BIM mRNA by binding to its 3’UTR and competitively inhibits the interaction between miR-221 and BIM, resulting in increased expression of BIM and promoting apoptosis in breast cancer cells (48). When DND1 is knocked down in breast cancer cells, it promotes the decline of BIM mRNA due to the increased binding of miR-221 to the Bim-3’UTR, thereby inhibiting apoptosis or leading to a poor prognosis in breast cancer patients. Conversely, DND1 protects BIM expression from miR-221 inhibition by competitive binding to BIM, thereby promoting apoptosis in breast cancer cells, but the expression level of DND1 is reduced in breast neoplasmss (48).

Zipcode Binding Protein 1 (ZBP1, also known as IMP-1 or IGF2BP1) belongs to a family of conserved RNA-binding proteins containing four HnRNP K (KH) structural domains and two RNA recognition motifs and is an mRNA regulatory factor (86). The expression of ZBP1 and β-catenin (associated with cell migration and proliferation) is synergistically regulated. ZBP1 binds to β-catenin mRNA *in vivo*, increasing the stability of β-catenin mRNA and inhibiting cell proliferation and migration. In metastatic breast cancer cell lines and tumors, the expression of ZBP1 is downregulated, leading to cell proliferation and migration (49, 50). Conversely, in breast cancer cells, IMP1 binds to the ACACCC motif of lncRNA UCA1 through the KH34 structural domain of the protein, destabilizing UCA1, promoting the decay of UCA1, and causing suppression of the UCA1-induced invasive phenotype (51). miR-122-5p is a suppressor of mRNAs associated with cell invasion, and UCA1 is a sponge for endogenous miR-122-5p. IMP1 binding to UCA1 destabilizes UCA1 and blocks the association between UCA1 and miR-122-5p, which in turn reduces the sponging effect of UCA1 on miRNAs, ultimately allowing the oncogenic effect of UCA1 to be diminished (51). IMP2 and IMP3 promote epithelial-mesenchymal transition (EMT) and metastasis, and they are overexpressed in TNBC. miR-200a, a family of tumor suppressor miRNAs, is downregulated in TNBC and maintains a stable epithelial phenotype by directly targeting the E-cadherin repressors ZEB1 and ZEB2, thereby significantly inhibiting EMT and metastasis (53). IMP2 and IMP3 are direct targets of miR-200a. IMP2 and IMP3 destabilize progesterone receptor (PR) mRNA by recruiting the CCR4-NOT transcriptional complex subunit 1 (CNOT1) complex and repressing miR-200a transcription. Overexpression of IMP2 and IMP3 repress miR-200a by post transcriptionally regulating PR mRNA stability to suppress miR-200a expression. Conversely, PR-induced miR-200a can also inhibit the expression of IMP2 and IMP3 by directly targeting their 3’UTR regions (53).

HuR is a tumor maintenance gene that allows malignant transformation, tumor growth and metastasis of RBPs.HuR binds to the 3’UTR of many proto-oncogenes and unstable AREs to regulate the stability and enhance the translation of target mRNAs, and it is also a key regulator affecting their translocation from the nucleus to the cytoplasm (87). Overall, in breast cancer cell lines, HuR has been shown to bind to mRNAs encoding 38 proteins that are associated with pathways of cell cycle arrest, angiogenesis and proliferation, and apoptosis, such as HuR, through stabilization of cell cycle protein-dependent kinase inhibitor 1 (p21), CDK1, CDK7, hypoxia-inducible factor 1α (HIF-1-α), calmodulin, vascular endothelial growth factor A (VEGF-A), MMP9, ER, HOX-A5, IL-8, FOXO1, CD9, CXCR-4, erbB2, SiRT1, and SOCS3, among other mRNAs, thereby increasing their protein levels, but HUR downregulates the mRNA levels of Wnt5a, tumor protein 63-delta Np63 (TP63), breast cancer type 1 susceptibility protein (BRCA1) and insulin growth factor 1 receptor (IGF1R) (54). Therefore, silencing and overexpression of HuR regulate the development of breast cancer.

LA-associated protein 7 (LARP7), a La family RNA-binding protein that controls RNAPII-suspended 7SK RNA, contains two RNA-binding domains: the RNA recognition motif (RRM) and the HTH La-type RNA-binding domain, which binds to and stabilizes 30 hairpins of 7SK RNA (the most abundant noncoding RNA in mammalian cells), forming the core of 7SK snRNP (7SK small ribonucleoprotein) (59). LARP7 is expressed at low levels in invasive breast cancer tissues and cells; therefore, when a reduction in LARP7 expression is observed, P-TEFb (positive transcriptional elongation factor b) in 7SK snRNP is released, and P-TEFb is reassigned to the transcriptionally active super elongation complex, allowing P-TEFb activation and EMT transcription factors (including FOXC2, Slug Twist1 and ZEB2) to be transcriptionally increased, which ultimately promotes breast cancer invasion, metastasis and EMT (60).

Tristetraprolin (TTP, also known as ZFP36) is an RNA-binding protein containing a tandem CCCH zinc finger structural domain and a proline-rich structural domain and a conserved carboxy-terminal sequence that normally binds to AU-rich elements (AREs) in the 3’-UTR of mRNA, causing the mRNA to depolymerize the poly(A) tail and leading to the degradation of its own mRNA (61, 62). In ERBB2 (oncogenic gene, also known as her2/Neu)-positive breast cancer, the RAS-MAPK kinase pathway is one of the signaling cascades activated by ERBB2 and synergizes with the PI3K/AKT pathway. The MAPK pathway stimulates TTP phosphorylation and becomes less active when it is phosphorylated, preventing deadenylation through the retention of 14-3-3 protein, thus failing to promote mRNA decay, leading to enhanced mRNA stability and translation and promoting the formation of cancer features, including proliferation, invasion, angiogenesis, metastasis and drug resistance (62).

Wig1 (also known as ZMAT3) is a direct target of the oncogene p53 and can encode a double-stranded RNA-binding zinc finger protein that inhibits cell proliferation by binding to p53 mRNA, stabilizing the AU-rich elements (AREs) in the 3’UTR of p53 mRNA and promoting its translation. p53 may also inhibit cell proliferation through Wig-1 by blocking HnRNP A2/B1, thus inhibiting cell proliferation through an unknown mechanism (63–65). Therefore, downregulation of Wig-1 may contribute to the development of breast cancer.

### RBPs regulate the poly(A) tail length of mRNAs of breast cancer-related genes through alternative polyadenylation

Alternative polyadenylation (APA) is an event leading to the formation of mRNA 3’ UTR isoforms that can produce shorter or longer mRNA isoforms by 3’-terminal cleavage and polyadenylation (CPA). The 3’ UTR was observed to be generally longer in breast cancer cells and is an important regulator of gene expression regulation (88). RBPs can also regulate the cleavage and CPA of target mRNAs by competing for or enhancing the binding of polyadenylation machinery proteins to their target sites, and thus, other auxiliary proteins, including RBPs, as well as polyadenylation machinery proteins strictly regulate polyadenylation (88).

Cytoplasmic polyadenylation element binding proteins (CPEBs), consisting of four paralogs (CPEB1-4) containing two zinc fingers and two RNA recognition motifs (RRMS), as well as a regulatory N-terminal region, are a family of RNA-binding proteins that directly mediate intracytoplasmic polyadenylation. They bind to target mRNAs through a mechanism of translational repression or cytoplasmic polyadenylation, allowing cytoplasmic polyadenylation elements (CPEs) to regulate the poly(A) tail length of mRNAs to regulate the translation of mRNAs (89). CPEB1 absence in breast cancer not only leads to the loss of polarity of mammary epithelial cells but also lengthens poly(A) and increases the polyadenylation and translation efficiency of MMP9 mRNA (tumor metastasis-promoting factor), which promotes the metastasis of breast cancer (66). CPEB2 plays a key role in the development of ER-positive breast cancer by regulating the poly(A) tail length of CPE-containing mRNAs, which in turn regulates the translation of mRNAs downstream of steroid hormone signaling, culminating in mammary gland development and ductal breast carcinogenesis (90). Overexpression of CPEB4 is associated with tumor growth, vascularization, migration, invasion and metastasis in breast cancer patients, causing an upregulation of Vimentin expression and promoting EMT, invasion and migration of breast cancer cells. However, the specific role and mechanisms of CPEB4 in breast cancer have not been fully investigated and reported in this regard (91, 92).

### RBPs affect breast cancer by regulating RNA subcellular localization

Nucleolin is a multifunctional RNA-binding protein (RBP) with multiple subcellular localizations, consisting of an amino-terminal charge region, a central region consisting of four RNA-binding regions, and multiple functional structural domains of a carboxy-terminal glycine/arginine (GAR) structural domain that drives subcellular localization mainly through the interaction of the protein with the kinesin light chain (93). In contrast, RBPs in numerous mammals have a GAR structural domain, which is a key determinant of the subcellular localization of the nucleolus, with implications for both their cellular function and disease-related occurrence (93). A few known cancer-associated ncRNAs interact with RBPs, such as AUF1, HuR, TTP, and IGF2BP1, which regulate ncRNA stability and subcellular localization in multiple ways (94).

HnRNPK is an abundant nuclear RNA binding protein in which lncRNA MLXIPL with a long internal exon containing multiple HnRNPK binding sites is strongly enriched in the nucleus of various human cell lines, and knockdown of HNRNPK strongly affects MCF7 cells (37, 38). In addition, a short sequence from the Alu element can bind to HnRNPK and increase its nuclear accumulation (37).

In addition to regulating the level of synthesis of specific proteins, CPEB1 coordinates the translational position of mRNAs through the regulation of their subcellular localization, while its regulated RNA localization is important for cell polarity. For example, CPEB1 mediates the apical localization of ZO-1 mRNA, a key tight junction component encoded by this mRNA in mouse mammary epithelial cells. This process is manifested by impaired colocalization of the tight junction protein ZO-1 and the tip protein syntaxin-3 and increased mislocalization of ZO-1 and the basal protein E-cadherin, ultimately leading to loss of cytosolic polarity in mammary epithelial cells, allowing epithelial-mesenchymal transition (EMT) and increased metastasis (66).

ZBP1 (IGF2BP1 or IMP1) acts as an RNA regulator associated with many cellular processes, including cell proliferation, cell polarity, induction of tumorigenesis and metastasis, binding to β-catenin (mRNA associated with cell proliferation and migration) to enable its activation, and leading to uncontrolled β-catenin by regulating the localization of β-actin mRNA. Disruption of β-catenin signaling allows the maintenance of cell polarity and directional movement, thereby inhibiting breast cancer cell chemotaxis and metastasis (49, 52). The extent to which IMP2 and IMP3 are involved in RNA localization is unclear, but IMP2 can bind to many nuclear-encoded mRNAs associated with mitochondrial function and may help localize transcripts to the mitochondria in a similar manner to that mediated by IMP1 and IMP3, transporting cytoskeletal and adhesion protein transcripts to the frontier of motile cells, while IMP2 binding to mitochondria regulates respiratory complex formation and facilitates oxidative phosphorylation (OXPHOS) (95). Therefore, we need to further investigate the functions of IMP2 and IMP3 in RNA localization in breast cancer and their mechanisms.

### Translational regulation of RBPs in breast cancer

Certain known RBPs (such as the splicing factors EFTUD2 and PRPF8) regulate different translational efficiencies by selective binding to 5’UTR structures; in addition, the use of other UTRs may expose the upstream ORFs of translation (UORF) or affect the stable binding sites of mRNA translation and/or miRNA (96). RBPs can facilitate mRNA translational control by recognizing the internal ribosome entry site (IRES) motif (a structural RNA element in the mRNA 5’UTR) and the translational (BAT) element activated by TGF-β in a cap-independent manner (97–99). Thus, RBPs are involved in various stages of translation, such as initiation, elongation and termination, and, concurrently, may bind to the 5’UTR or 3’UTP to regulate translation efficiency.

HnRNP E1 can regulate the translation of specific proteomes directly or indirectly by binding to RNA: (1) binding of HnRNP E1 to specific targets, which directly inhibits translation by preventing translation elongation; (2) relying on selective splicing; and (3) positively regulating translation by binding to the 3’UTR of transcripts (31). In particular, the ribonucleoprotein (MRNP) complex binds to the 33-nucleotide TGFβ-activated translation element (BAT) in the 3’UTR of the mRNA, thereby silencing the translation of the mRNA encoding the mesenchymal protein. HnRNP E1 is a key component of the BAT-binding mRNP complex (31). In addition, HnRNP E1 can prevent the release of eEF1A1 from the ribosomal A site after GTP hydrolysis by binding to the 3’UTR BAT element of eukaryotic elongation factor-1A1 (eEF1A1), bringing translation elongation to a halt and leading to translational silencing of the two EMT transcripts DAB2 and ILEI (97, 98). TGFβ activates a nonclassical kinase cascade reaction that induces protein kinase BB/Akt2-mediated phosphorylation of HnRNP E1 at serine 43, resulting in release of the mRNP complex from the BAT element and restoration of translation (32). Both TGFβ stimulation and silencing of HnRNP E1 in breast cancer increase the translation of ILEI (oncogenic factor associated with EMT and tumorigenesis), which mediates signaling through STAT3, thereby inducing the formation of BCSCs (breast cancer stem cells) and promoting EMT (33).

HuR may accelerate the initiation of mRNA translation by binding to the 3’-UTR of the target mRNA through interaction with eIF3a (a subunit of the eukaryotic translation initiation factor 3 complex) to regulate protein synthesis (55). HuR not only promotes the translation of p53 mRNA directly but also increases p53 protein synthesis by blocking UV-induced miRNA miR-125b, which has the effect of inhibiting p53 translation (56). In addition, HuR both stimulates XIAP IRES activity and promotes translation of endogenous XIAP mRNA, resulting in elevated levels of XIAP protein and achieving enhanced cytoprotective effects. XIAP is a member of the endogenous inhibitor of apoptosis (IAP) protein family (99). Taken together, HuR may regulate the efficiency of translation through binding to the corresponding breast cancer target mRNA 3’UTR or 5’UTP, which in turn regulates the development of breast cancer.

Eukaryotic translation initiation factor 4E (EIF4E), one of the components of the translation initiation complex EIF4F, recognizes and binds the m^7^G cap at the 5’ end of mRNA and is a key factor in initiating translation, while its phosphorylation increases mesenchymal markers such as N-calmodulin, wave proteins and fibronectin, which in turn promote tumor invasion, EMT and metastasis (67). When the mRNA unravels, ribosomes are recruited into the mRNA, and translation begins. Overexpression of EIF4E in cancer elevates c-MYC and Cyclin D1 protein levels, which promote proliferation and inhibit apoptosis. Because of the low abundance of eIF4E, it is suggested that it plays a role in translation by regulating the efficiency of mRNA translation (67, 68). The phosphorylation of EIF4E is regulated to some extent by MAP kinase integrated kinase MNK1/2 at serine 209, so the phosphorylation of EIF4E can be blocked by MNK inhibitors. Simultaneously, the synthesis of Cyclin D1 is reduced, and the proliferation and metastasis of breast cancer cells are inhibited by MNK inhibitors (69, 70).

The ACHN gene (also known as La-associated protein 6; LARP6) is a protrusion-rich RNA-binding protein that is also enriched in translation initiation and elongation factors in front of the protrusion and is a key point of translation for local ribosomal protein-encoding mRNAs (RP-mRNAs), promoting migrating cell RP synthesis, protein synthesis and ribosome biogenesis. In human breast cancer, LARP6 overexpression is associated with epithelial-to-mesenchymal transition (EMT) (57, 58).

## Function of RBPs in breast cancer

**Figure 2:**
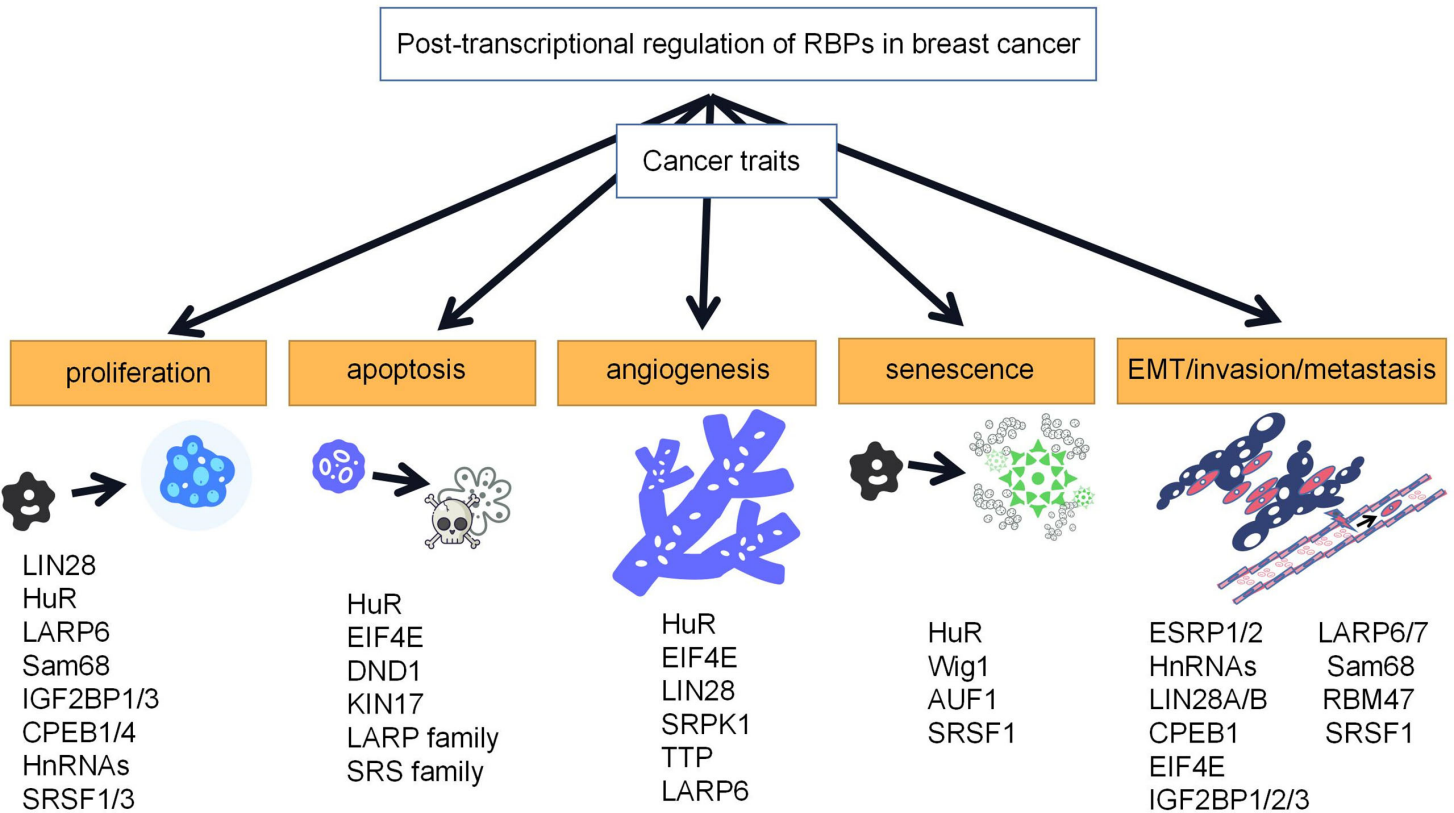
Roles of RBPs in breast cancer. RBPs play important roles in proliferation, apoptosis, angiogenesis, senescence, and EMT/invasion/metastasis of breast cancer. Representative RBPs for breast cancer traits are listed in the schematic diagram.

The occurrence of breast cancer may be associated with many factors, including genetic and environmental factors, and RBPs can be involved in the development of breast cancer by regulating the expression levels of proto-oncogenes and oncogenes. Aberrant expression of these RBPs can affect every stage of breast cancer, including proliferation, apoptosis, angiogenesis, senescence, and EMT/invasion/metastasis, and thus, their roles are complex and diverse (**Figure 2**).

### RBPs play a proliferative role in breast cancer cells

Most of these RBPs are associated with cell proliferation, and excessive and abnormal proliferation is key to the development of cancer and may gradually evolve into malignancy.

It has been shown that in breast cancer cells, aberrant activation of LIN28 not only represses let-7 to enable it to function as an oncogene but also promotes and maintains the proliferation of breast cancer cells by directly or indirectly stimulating the expression of tumor growth-related genes (including HER2 and HMGA1) after transcription (16, 100).

The HnRNP family of HnRNPA1 and HnRNPI were reported to be overexpressed in breast cancer and to regulate selective splicing of PKM to promote tumor cell proliferation (25). In contrast, HnRNP D, also known as AUF1, controls tumor proliferation by regulating the translation level of Myc mRNA (101). Overexpression of HnRNP K in breast cancer cells significantly increases target c-myc promoter activity and c-Myc protein and HnRNP K protein levels and promotes breast cancer cell proliferation in a nondependent anchoring manner (38).

IGF2BP1 inhibits cell proliferation by regulating the targets of mRNAs associated with breast cancer, such as binding to β-catenin mRNA and improving its stability (49, 50). IGF2BP3 accelerates the proliferation of breast cancer cells not only by regulating the target of the corresponding mRNA but also by competitively binding with miR-3614-3p to the 3’UTR of the host gene TRIM25 and protecting TRIM25 mRNA from miR-3614-mediated degradation (102).

CPEB1 regulates the translation of CPE-containing mRNAs by regulating their poly(A) tail length, thereby affecting cell proliferation (90). CPEB4 is overexpressed in breast cancer cells and alters the proliferative state of the tumor by affecting the expression level of its target mRNA (91, 92).

HuR promotes breast cancer proliferation through mRNAs that regulate the cell cycle or proliferation-related genes and pathways, such as CDK2 and Cyclin E1 (54, 103). LARP6, an oncogene, is highly expressed in myoepithelial cells and mammary basal cell-like invasive ductal carcinoma of the breast and is also aberrantly expressed in MDA-MB-231 breast cancer cells, promoting cell proliferation (104).

Sam68 can promote cell proliferation by regulating the selective splicing of multiple genes, such as Bcl-xL, Cyclin D1, and CD44 (46). In breast cancer, Sam68 is overexpressed, and acetylation of Sam68 and enhancement of its binding to poly(U) RNA by the acetyltransferase CBP can exert a proliferative effect on tumor cells when acetylation of Sam68 and enhancement of RNA binding activity are present (105).

The splicing factor SRSF1 is upregulated in human breast tumors and acts as a target involved in gene expression regulation, cell cycle and proliferation control, as well as cell death and survival, such as through selective splicing (AS). Overexpression of one such heterodimer, exon 9, including CASC4, promotes an increased follicle size and proliferation (106). In addition, the TDP43/SRSF3 complex controls specific splicing events, and TDP43 (TAR DNA-binding protein 43) is an important splicing regulator; when the TDP43 or SRSF3 gene is knocked out, reduced proliferation of mammary epithelial cells is mediated by splicing regulation of Numb exon 12 (107).

### Role of RBPs in apoptosis of breast cancer cells

Cancer cells have the ability to not only continuously proliferate but also prevent cell death. Normal cells undergo apoptosis; however, cancer cells perpetually evade apoptosis, thus maintaining the activity of cancer cells and promoting further tumor development. Some of these RBPs are involved in this anti-apoptotic effect by regulating apoptosis-related mRNAs in breast cancer target cells, such as Myc, Mcl-1, p53, Bcl-2 and other mRNAs (108, 109).

HuR affects apoptosis in breast cancer cells by regulating mRNAs that stabilize anti-apoptotic genes, such as mRNAs for p53, bcl-2, Fas, and TNF (54–56). HuR can also influence the anti-apoptotic effects of cells by stimulating XIAP IRES activity and promoting the translation of endogenous XIAP mRNA (99).EIF4E is involved in regulating the expression levels of c-Myc and Bcl-xL to influence apoptosis (110). DND1 expression is downregulated in breast cancer cells and is associated with a poor patient prognosis, and it promotes apoptosis by inducing BIM mRNA expression through competitive interactions with miR-221 (48). In breast cancer, downregulation of KIN17 inhibits cell proliferation and promotes apoptosis, which is associated with increased Caspase3/7 activity (111). The LARP family affects cell growth by controlling the stability of cell survival genes (e.g., Bax and Bcl-2) (104). The SRS family affects apoptosis mainly by regulating the selective splicing of tumor-associated genes (such as BIM and BIN1) (39, 78).

### RBPs affect angiogenesis in breast cancer

Both normal cells and cancer cells need oxygen and nutrients, especially cancer cells, which need larger amounts. The process of cancer cell metastasis requires passage through blood vessels, so angiogenesis is necessary for tumor development. Angiogenesis is promoted by angiogenic activators, such as vascular endothelial growth factor, tumor necrosis factor-α, and hypoxia-inducible factor-1α (HIF-1α) (112). RBPs are involved in regulating the expression of angiogenic factors and play an important role in tumor progression.

HuR is involved in regulating the expression of several angiogenesis-related genes, including vascular endothelial growth factor α (VEGFα), HIF1α and platelet response element 1 (TSP1), a known anti-angiogenic gene. Surprisingly, overexpression of HuR in ER-breast cancer leads to an increase in TSP1 and a decrease in VEGF expression, resulting in reduced tumor angiogenesis, so the exact mechanism of the antiangiogenic effect against HuR is not fully understood but may involve an interaction between HuR and microRNAs (113). EIF4E may be an important regulator of angiogenic factor (such as IL-8 and VEGF) production in breast cancer cells, affecting angiogenesis by regulating the translation of its target mRNAs (VEGF, Cyclin D1 and FGF2), and is associated with a poor prognosis in breast cancer (114, 115). LIN28 affects angiogenesis by regulating the expression level of let-7d (116). In breast cancer, SRPK1 can mediate SRSF1 phosphorylation and promote angiogenesis by regulating VEGF premRNA splicing to generate proangiogenic isoforms (117). Regulation of the mRNA half-life plays an important role in breast cancer. TTP, an RNA-binding protein 1 and KH-type splicing regulatory protein that normally promotes mRNA degradation, reduces the half-life of VEGF mRNA and slows the growth of RAS-transformed cell-derived nude mouse xenograft tumors, in turn reducing the microvessel density in tumors and leading to the inhibition of tumor growth and angiogenesis (118). LARP6 is overexpressed in breast cancer and promotes angiogenesis by upregulating the expression of MMP-9 and VEGF (57, 104).

### Role of RBPs in the senescence of breast cancer

Cellular senescence is a biological process influenced by multiple factors that can lead to permanent cell cycle arrest. RBPs can lead to abnormal gene expression during cellular senescence, which in turn regulates the senescence of tumor cells.

In immortalized MCF-10A mammary epithelial cells, HuR can specifically bind to two U-rich elements in the 3’UTR of p63 mRNA, which in turn downregulates the expression level of the tumor suppressor △Np63 and slows cellular senescence (119). Wig1 promotes the degradation of p21 mRNA by binding to the stem–loop structure near the miRNA target site, thereby reducing the expression of p21 and inhibiting cellular senescence (120). AUF1 inhibits the senescence of breast cancer cells by participating in the degradation of the senescence-related genes p16, p53, and p21 (121). SRSF1 stabilizes p53 by recruiting the RPL5-MDM2 complex and increases p53 protein expression and activity, leading to premature cellular senescence (122).

### RBPs and breast cancer EMT with invasion and metastasis

During cancer development, RBPs can promote EMT in tumors through various regulatory mechanisms, and when EMT occurs, they inhibit intercellular adhesion and cell polarity, which also promote cancer invasion and metastasis.

ESRP1 and/or ESRP2 further promote EMT by regulating the selective splicing of Rac1 and CD44. In breast cancer, the reduction of ESRP1 changes the variant expression of CD44v from CD44v to CD44, thus inhibiting its metastasis in the lung (42, 43). In addition, HnRNP M can promote the expression of mesenchymal-specific CD44v through competitive interaction with ESRP1, thereby promoting breast cancer metastasis (25, 34).

Members of the HnRNP family can promote EMT and tumor invasion and metastasis. HnRNP E1 regulates the splicing of EMT-related genes and silences their translation in a TGF-β-dependent manner by binding to C-rich elements in the 3’UTR of certain mRNAs, including CD44 and PNUTS. In normal mouse mammary epithelial cells (NMuMG), when HnRNP E1 is silenced, it increases migration and invasiveness *in vitro* and promotes the formation of distant metastases *in vivo* (123). In breast cancer, HnRNP-K is highly expressed and promotes metastasis by inducing the extracellular matrix, cell motility, angiogenesis-related genes and invasive signaling pathways, such as the regulation of cell migration *via* the Ras/MEK/ERK-MMP-3 pathway (124). HnRNP A1 affects the expression of SREBP1, suppresses E-cadherin, and promotes formation of the Snail/HDAC1/2 complex by regulating the processing of miRNA-18a (pri-mir-18a), leading to EMT in breast cancer cells (23).

Overexpression of LIN28A/B is associated with breast cancer tumor migration and invasion, and the mechanism may be related to the let-7 gene (17, 18). In normal mouse mammary epithelial cells (NMuMG), KHSRP can inhibit TGF-β-mediated EMT by activating miR-192-5p, thereby reducing EMT-associated factors (20). CPEB1 is negatively associated with breast cancer metastasis, and mechanistically, knockdown of CPEB1 can contribute to breast cancer metastasis through polyadenylation and translation of MMP9 mRNA (66).In breast cancer, EIF4E increases mesenchymal markers by regulating its phosphorylation, which in turn promotes tumor EMT, invasion and metastasis (67).

IGF2BP1 binds to target mRNAs, such as β-catenin or lncRNA UCA1, by regulating their stabilization and localization, thereby inhibiting metastatic cell invasion and migration, but IGF2BP1 is expressed at low levels in metastatic breast cancer (49–51). In triple-negative breast cancer, IGF2BP2 and 3 contribute to cell migration and invasion by recruiting the CNOT1 complex to destabilize PR mRNA and thereby synergistically promote cell migration and invasion (53).

LARP6, a member of the La-associated protein (LARP) family, is aberrantly expressed in MDA-MB-231 breast cancer cells, resulting in a series of physiological responses with enhanced invasive behavior in *in vitro* and *in vivo* xenograft models, including proliferation, platelet pseudopod formation, EMT, invasion, MMP-9 and VEGF expression, angiogenesis and tumor growth (57). LARP7 is expressed at low levels in breast cancer; therefore, elevated levels of this protein are associated with overall improvement and longer recurrence-free survival. It has been found that short hairpin silencing of LARP7 in MCF10A cells can upregulate the expression levels of P-TEFb-mediated EMT and metastatic genes (such as Slug, Twist1 and ZEB2), thereby promoting tumor invasion and metastasis (104).

It has been shown that Sam68 can induce the BRK/ERK5/Sam68 complex through the activation of Met receptors (and ErbB receptors), which function to reprogram cellular mRNA splicing, thereby promoting protein expression and ultimately favoring breast cancer cell migration (45). RBM47 inhibits tumor progression and metastasis by increasing the secretion of DKK1, which in turn inhibits tumor progression and metastasis (47). SRSF1 promotes EMT, invasion and migration of breast cancer by generating the expression of splice variants lacking the BH3 structural domain (39).

## RBPs as biomarkers of breast cancer and their future development prospects for clinical treatment

With the in-depth study of RBPs in breast cancer in recent years, there is a new understanding of their function and mechanism in regulating RNAs, which are closely related to breast cancer proliferation, invasion, metastasis, MET and drug resistance.

### RBPs as biomarkers and potential therapeutic targets for breast cancer

Through a large amount of clinical data and literature in recent years, it has been shown that many RBPs can serve as biomarkers and potential therapeutic targets for breast cancer. For example, CPEB4, which is overexpressed in breast cancer, can induce MET and metastasis in breast cancer cells and may become a potential molecular marker for treatment and prognosis prediction in advanced breast cancer (91). It has been shown that DND1 can inhibit the binding of miRNAs to BIM in breast cancer cells and highlighted that DND1 can promote apoptosis in breast cancer cells; thus, DND1 may be a potential therapeutic target for breast cancer (48). It has also been found that NONO is a key regulator of breast cancer proliferation, regulating the expression of the cell proliferation-related genes Skp2 and E2F8 at the posttranscriptional level, and it may become a new diagnostic marker and therapeutic target for advanced breast cancer (125). In addition, the RNA-binding protein PSF promotes the proliferation of ER-positive breast cancer cells by regulating the expression of ERα, TRA2B, aberrant spindle-like microcephaly associated protein (ASPM), and SEC1 family structural domain 2 (SCFD2) mRNAs at the posttranscriptional level, and it may be a potential diagnostic and therapeutic target for hormone-resistant breast cancer and primary breast cancer, as well as a potential poor prognostic factor for ER-positive breast cancer (126). According to an experimental validation, downregulation of the expression of three RBPs (MRPL12, MRPL13 and POP1) resulted in significant inhibition of breast cancer cell survival and migration *in vitro*, suggesting their potential to be designed as biomarkers and/or therapeutic targets for breast cancer (127). There are data supporting that Sam68 is overexpressed in human breast cancer cell lines and tissues and may play an important role in promoting proliferation and cell cycle progression in human breast cancer, so sam68 could be used as a prognostic or diagnostic biomarker for breast cancer treatment. Silencing sam68 plays an antiproliferative role, mainly through activation of the FOXO/p21/p27 pathway and inactivation of the Akt/GSK-3β signaling pathway, so it is also a potential target for the future treatment of breast cancer (46). Finally, EIF4A3, an important RBP, is overexpressed in breast cancer and regulates the cell cycle by binding to SEPT9 premRNA to promote circ-septin 9 (SEPT9) expression, so it may also serve as a diagnostic marker or therapeutic target for breast cancer (128, 129).

### Therapeutic approaches for cancer RBPs and future development directions

**Figure 3:**
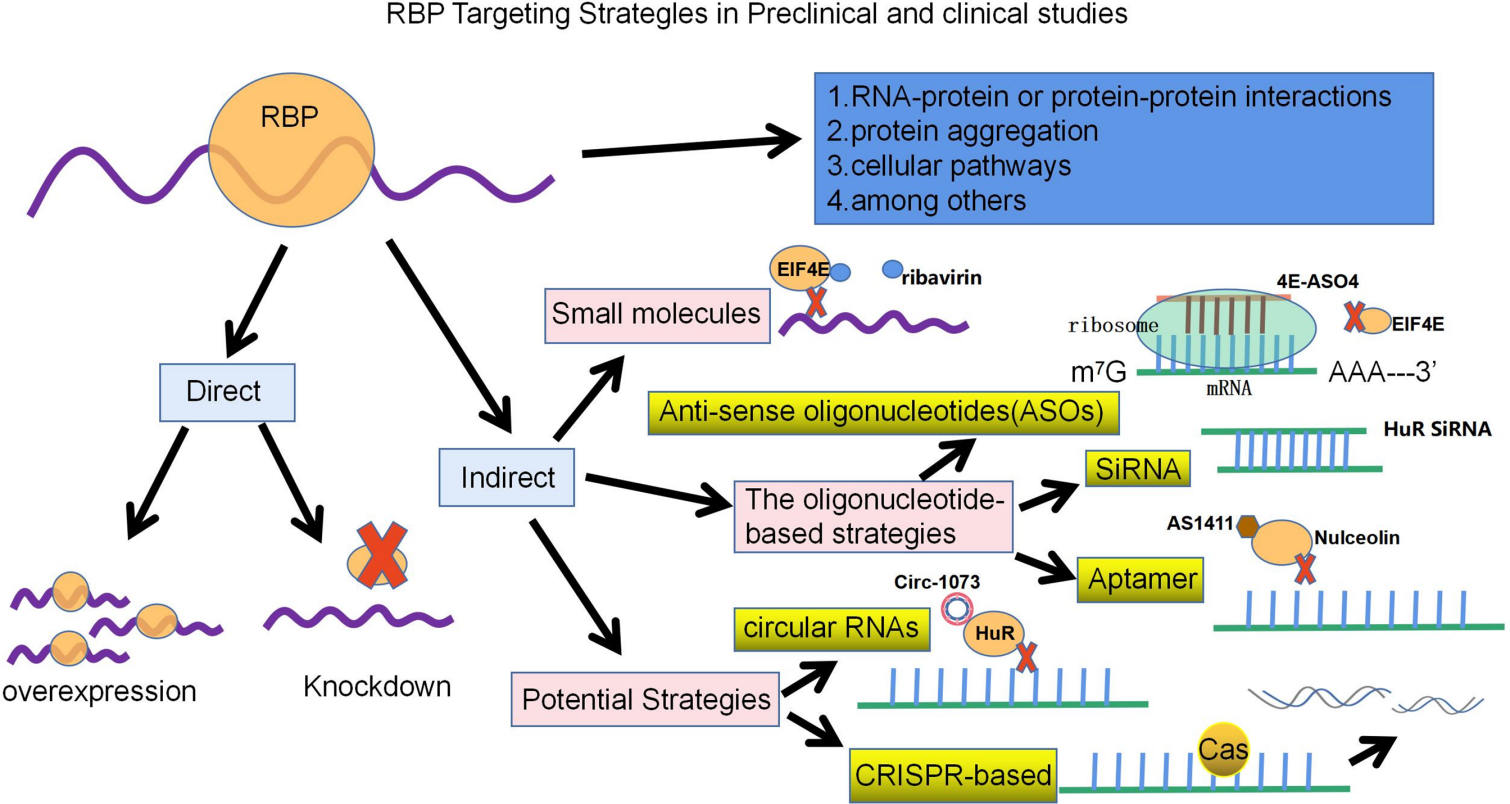
With the development of Clip-sequencing, a technique to identify genome-wide RNA binding motifs *in vivo*. These strategies may involve RNA-protein or protein-protein interactions, cellular pathways and protein aggregation, among others. Direct therapeutic strategies revolve around knocking down or overexpressing specific RBPs, while indirect approaches, on the other hand, include the use of small molecules, oligonucleotide-based strategies (ASO, SiRNA, Aptamer) and other potential strategies. The schematic diagram lists some of the RBPs that have made breakthroughs in breast cancer treatment strategy research.

Previous reports have shown that RBPs play an important role not only in the expression of normal cells but also in the regulation of breast cancer development. In recent decades, there have been no specific drugs directly targeting RBPs for treatment, but recent developments have revealed that we can target RBPs directly or indirectly with a variety of different approaches. These strategies may involve RNA–protein or protein–protein interactions, cellular pathways, and protein aggregation, among others. Direct therapeutic strategies revolve around the inhibition or overexpression of specific RBPs, while indirect approaches include the use of small molecules, oligonucleotide-based strategies, oligonucleotide aptamers, synthetic peptides and other potential strategies for targeting RBPs in cancer, with the use of small molecules being the most common strategy for targeting RBPs (**Figure 3**) (7, 130).

Small molecule drugs can target RBP function in various human diseases, including breast cancer, and have been clinically tested and reported to have anticancer effects. Small molecules can be used to inhibit RBP function in breast cancer by binding to RBD. Taking EIF4E as an example, the binding of EIF4E to the cap structure is used as a target (7). Ribavirin, a guanosine ribonucleoside analog, was initially found to mimic the cap structure and subsequently compete with endogenous mRNA for binding EIF4E, blocking the transport and translation of EIF4E-regulated oncogenes (such as Cyclin D1) to reduce tumor formation *in vivo* and *in vitro* (131). It has shown good preclinical efficacy and potential efficacy in clinical trials in metastatic breast cancer (132). In addition, use of the N-7 benzylguanosine monophosphate tryptamine phosphoramidate prodrug (4Ei-1) prevents EIF4E cap binding and triggers proteasomal degradation of EIF4E, thereby sensitizing breast cancer to gemcitabine chemotherapy (133). Several small molecules, 4EGI-1, 4E1RCat and 4E2RCat, have been designed to disrupt the interaction between EIF4E and EIF4G to inhibit cap-dependent translation and promote apoptosis of tumor cells *in vitro* and *in vivo*, with significant antitumor effects, especially in breast cancer xenograft models (7, 134).

Another anticancer strategy involves the use of oligonucleotide-based strategies, including short-stranded antisense oligonucleotides (ASOs), small interfering RNAs (SiRNAs), and aptamers. ASOs can disrupt protein production by blocking ribosome binding to inhibit translation of target mRNA or binding to RNA *via* Watson-Crick base-pairing, which in turn promotes the degradation of target RNA (*via* RNAase H-mediated degradation), altering RNA metabolism, or upregulating the expression levels of certain genes; therefore, therapeutic ASOs are considered a promising approach for targeted treatment of TNBC (7, 135). For example, in breast cancer mouse transplant tumors, the second-generation antisense oligonucleotide 4E-ASO4 inhibits EIF4E by modifying it to provide nuclease resistance, shows its antitumor activity and is well tolerated with no adverse effects on liver function or body weight (136). SiRNA-based therapies involve the introduction of synthetic SiRNAs encapsulated in nanocarriers into target cells to induce RNAi, thereby inhibiting the expression of specific mRNAs. Thus, SiRNA-mediated gene silencing effects are produced by directing the degradation of specific mRNAs (135). The SiRNA of HuR was loaded into folic acid (FA)-coupled nanoparticles, and the formulation was found to be effective in reducing HuR expression and cell proliferation and to synergistically enhance antitumor effects with reduced cytotoxicity. Furthermore, HuR silencing sensitizes triple-negative breast cancer cells to radiation therapy due to its ability to induce oxidative stress and DNA damage (7). In addition to HuR, SiRNA against EIF4E not only inhibits growth and promotes apoptosis in human breast cancer cells, but also enhances the cytotoxic effect of cisplatin (137). Aptamers can fold into sequence-specific three-dimensional structures that can recognize their unique targets and have antibody-like functions (7). The aptamer AS1411 (formerly known as AGRO100), targeting RBP nucleolin, is a 26-nucleotide DNA-based aptamer that forms a stable G-quadruplex structure that is resistant to nucleases and was the first aptamer to be used in cancer clinical trials. Nucleolin regulates several essential cellular processes, namely, RNA polymerase I transcription, proper folding of mature and prethoracic RNA, mRNA translation, and mRNA stability, and it is overexpressed in cancer (138, 139). AS1411 binds to the external structural domain of the nucleolus and inhibits tumor growth in *in vitro* and *in vivo* xenograft models of breast, lung and kidney cancer (139).

Other potential strategies to target RBPs for the treatment of breast cancer include circRNAs and CRISPR-based therapies. circRNAs act as miRNAs or RBP sponges in cancer, altering gene expression levels by regulating transcription and splicing and acting as translation templates. Some circRNAs can also induce the proliferation and progression of TNBC by regulating the transcription of tumor-associated signaling pathways and related genes (140). For example, circ-1073 binds to and increases the expression of HUR, which in turn increases the levels of cleaved Caspase3/9 and E-cadherin, thereby suppressing the malignant biological behavior of breast cancer (141). Interestingly, a circRNA may contain several loci of one or more RBPs, thus regulating the function of RBPs by acting as an RBP sponge or decoy (142). In the last decade, development of the clustered regularly interspaced short palindromic repeat sequence/CRISPR-associated protein 9 (CRISPR/CAS9) system has also had potential therapeutic applications in cancer therapy. CRISPR can directly target RBPs or their functions in different ways. For example, it can be used to knock down oncogenic RBPs in cancer cells, regulate RBP binding sites in mRNAs, or correct cancer-specific RBP mutations that lead to abnormal splicing of oncogenes (7).

In summary, some therapeutic strategies are still in preclinical and clinical trials for evaluation, and we have a lot of work ahead, so the development of a new therapeutic strategy is long and needs to be supported by expansive clinical data.

## Conclusions

With the in-depth study of gene expression abnormalities in cancer and our further understanding of posttranscriptional regulation in cancer, there is a strong interest in RBPs because of their involvement in all aspects of posttranscriptional regulation, including mRNA processing, RNA stability, alternative splicing, alternative polyadenylation, subcellular localization and translation, emphasizing that they play an important role in cancer development. As described in this review, certain RBPs collectively regulate multiple genes in breast cancer through multiple functions, leading to different progression and changes in cancer and, for this reason, to the design of new diagnostic and prognostic biomarkers with potential targets for new therapeutic approaches, allowing us to detect breast cancer earlier and develop rational prognostic treatment strategies.

To summarize, dysfunction of RBPs and consequent abnormalities in posttranscriptional gene expression may contribute to breast cancer development and progression. Although in recent years, a large number of researchers have tried to target RBPs and/or their chaperones in preclinical and clinical studies using small molecules, SiRNAs, ASOs, aptamers and nanoparticle carriers of peptides, only a few RBPs have been used in cancer therapy. Because of the large number of RBPs associated with cancer and the lack of available structure-function studies to predict these targets bioinformatically, there is still a long way to go regarding the development of therapeutic strategies against RBPs.

With the development of in-depth research techniques, such as Clip-sequencing(HITS-Clip), PAR-Clip, RIP-SEq and iCLIP, we have discovered many new RBPs and their partners and conducted functional studies. However, the complexity of interactions between RBPs and other cellular networks, pathways and disease-related processes and the function of RBPs are not incompletely understood and under investigation, thus limiting the associated therapeutic strategies associated. In conclusion, our understanding of RBPs related to breast cancer is still in the initial stage, and a large amount of additional research is needed. It is hoped that RBPs will become an important means of clinical treatment of breast cancer in the future.

## Author contributions

XL and JZ searched the literature and wrote the manuscript. LL,WZ,SZ, LC, YW, HZ and JW searched the literature. FG and WC conceived the idea for the review, critically revised the manuscript and provided the final approval. All authors contributed to the article and approved the submitted version.

## Funding

The present study was supported by the National Natural Science Foundation of China (grant nos. 82060543 and 82060538), the Yunnan Fundamental Research Projects (grant no. 202101AT070347) and The Yunnan Fundamental Research Projects (grant no. 202201AT070119).

## Conflict of interest

The authors declare that the research was conducted in the absence of any commercial or financial relationships that could be construed as a potential conflict of interest.

## Publisher’s note

All claims expressed in this article are solely those of the authors and do not necessarily represent those of their affiliated organizations, or those of the publisher, the editors and the reviewers. Any product that may be evaluated in this article, or claim that may be made by its manufacturer, is not guaranteed or endorsed by the publisher.
